# Peasant family farming as an alternative to industrial agriculture: Implications for maternal and child health in Paraguay

**DOI:** 10.3389/fepid.2022.954081

**Published:** 2022-11-29

**Authors:** Stela Benitez Leite, Babak Tofighi

**Affiliations:** ^1^Department of Pediatrics, School of Medicine, National University of Asunción, San Lorenzo, Paraguay; ^2^Department of Population Health, New York University School of Medicine, New York, NY, United States; ^3^Division of General Internal Medicine, Bellevue Hospital Center, New York, NY, United States

**Keywords:** developmental toxicology, toxicity testing, exposure, pesticide, neurodevelopment, industrial agriculture, family agriculture

## Introduction

The World Health Organization (WHO) and the United Nation's Food and Agriculture Organization established food system sustainability as a critical goal for improving nutritional and health outcomes (1). However, present industrialized agricultural practices are exacerbating environmental degradation and public health outcomes globally.

In Paraguay, industrial agriculture is eroding biodiversity and contaminating surface water and aquifers (2), particularly in peasant and indigenous communities (3). Peasants are also being displaced to urban areas where the proportion of urban residents in Paraguay has nearly doubled between 1970 (37.4%) and 2021 (62.5%), and anticipated to continue rising (4). Lastly, industrial agriculture has accelerated a dietary shift to processed foods laden with sugars, fats, and preservatives (5).

Achieving food system sustainability requires a major shift in agricultural practices to ensure access to diverse and healthy foods, particularly for maternal and child health. Transitioning from industrial to sustainable agriculture systems also reduces early life exposure to synthetic chemical pesticides and fertilizers in early life. According to the International Agency for Research on Cancer, numerous pesticides have been categorized as being potentially carcinogenic for increasing genotoxic, endocrine, and chromosomal anomalies.

## Environmental toxins and child health

The developing nervous system during early childhood is especially vulnerable to environmental toxins. A positive dose-response relationship to pesticide exposure, including endocrine disrupting chemicals, and neurodevelopmental outcomes have been observed, ranging from cognitive deficits (e.g., reduced working memory), behavioral deficits (e.g., reduced attention), motor deficits (e.g., abnormal reflexes), and psychiatric outcomes (e.g., depression, suicide) (6).

Maternal pesticide exposure is especially hazardous due to its negative impact on the mother, among children exposed during breastfeeding, and its teratogenic effect on gamete and embryo development, which undergo reprogramming in humans between weeks 4 and 12 of gestation (7). The multitude of chemical compounds and proteins naturally modifying the DNA, also known as the epigenome, are also at risk of being modified following chemical exposures. Such epigenetic alterations include changes to the DNA, histone protein damage, that serve as the scaffolding for the genome, and non-coding RNA modifications that regulate gene expression. As a result, variations to the epigenome can result in changes to the phenotype without a change in the genotype (7, 8).

Generational toxic exposures have been shown to promote transgenerational epigenetic inheritance of adult-onset diseases. For instance, in rat models exposed to glyphosate, transgenerational pathologies consisted of obesity, diseases of the prostate, kidney, and ovaries, and birth abnormalities, particularly among the offspring generation (F2) and in transgenerational great-grandchildren (F3); (9). More recent human studies exploring glyphosate exposure suggest potential cytotoxic and genotoxic effects, and impaired immune system and cognitive functioning (10).

Among rural peasant children in Paraguay, scientific evidence suggests greater exposure to intensive fumigation due to industrial agriculture that may be associated with increased genotoxic and cytotoxic effects compared to other populations without pesticide exposure (11). As a result, our team and others throughout Latin America have noted increased prevalence of cancer, diabetes, respiratory diseases, neurodegenerative and neurodevelopmental disorders among children that may possibly be associated with chronic pesticide exposure (11–13). Childhood exposure may result from pesticide drift due to residing in proximity to industrial soybean farms and/or excessive fumigation by family members with little training in the use and effects of such chemicals in home cultivations (14, 15).

## Improving the nutritional status of rural children

Scaling of sustainable and equitable agricultural practices remains a critical source of diverse and nutritious food and is directly linked with improvements in the nutritional status of rural children in Paraguay (16). In a study of rural peasant children, our team observed no evidence of chronic malnutrition, hypoproteinemia, or anemia, and most demonstrated adequate height for age [93%; (11)]. However, nationwide findings presented by the Paraguayan National Institute of Food and Nutrition (INAN) in 2020 revealed a high percentage of children experiencing chronic malnutrition (12.7%), acute malnutrition (5.6%), and obesity (7.2%) highlighting the need to increase access to nutritious foods (17).

Expanding sustainable agricultural practices in rural regions is especially difficult in Paraguay as it remains one of the most inequitable countries in Latin America with respect to land ownership (18). Although the national government has committed to the 2030 United Nations Sustainable Development Goals, the likelihood of mitigating pesticide use and improving the nutritional status of children seems unlikely. Concerns for food security, particularly among indigenous communities, children, women, and older adults, have been exacerbated in the context of a worsening health, climatic and social crisis (19).

In addition, unregulated industrial farming practices centered on transgenic soybean exports has resulted in mass deforestation, excessive use of hazardous agrochemicals, disruptions to sustainable family farming practices, and displacement of rural communities to urban settings, which has further deteriorated the nutritional status of rural children (20, 21). Between 2002 and 2017, peasant family farming decreased by 33% nationally. However, regions with a higher percentage of industrial agriculture experienced more dramatic reductions in peasant family farming (64%) (22). For instance, in the state of Caazapá, which experienced a dramatic shift to industrial agriculture, crop yields for tomatoes, carrots, and other vegetables decreased by 98% between 2002 and 2017 (23).

## Improving access to affordable and nutritious food among urban children

The homogenization of dietary preferences in mostly urban regions in Paraguay has contributed to the increased consumption of ingredients that exceed recommended limits on sugar, saturated fat, and sodium intake established by the Pan American Health Organization (24). Such dietary trends are concerning and associated with increases in obesity, diabetes, and other chronic diseases that the existing health system is unable to manage (25). A recent survey of Paraguayan residents revealed that roughly 17% of food consumed in Paraguayan cities by the urban poor are produced by local subsistence farming compared to 48% of foods consumed by low-income rural residents. While 27% of consumed foods in Paraguay are categorized as processed foods, urban residents consume 10% more processed foods relative to their rural counterparts.

Lastly, Paraguay imports nearly half of its consumed fruits and vegetables and highlights the need for scaling peasant subsistence farming to improve the nutritional status of both urban and rural residents (21). Not surprisingly, a key recommendation to the Paraguayan State by the United Nations Special Rapporteur for the Right to Food emphasizes the need to “protect and promote family farming as a productive model” and “supporting small-scale food producers, in particular women, indigenous peoples and youth to ensure equitable access to land and other productive resources” (19, 26).

## Conclusion and recommendations

The impact of chronic pesticide exposure and poor access to affordable and nutritious food remains a global public health challenge. Findings in Paraguay highlight the importance of ensuring nutritious food access derived from sustainable peasant family farming practices, particularly for children exposed to pesticides. Multi-pronged approaches to improving rural child health should include: (1) supporting peasant family farming to offset the ecological and public health collapse associated with industrial agriculture; (2) establishing and monitoring worldwide standard values for pesticides in soil, air, and drinking water; (3) expanding studies on the immediate and longitudinal hazards of chronic pesticide exposure as the unregulated use of carcinogenic and endocrine-disrupting compounds increases, particularly in low- to middle-income countries; (4) improving peasant education to improve pesticide management and safety; and (5) increasing access to affordable and nutritional foods in urban settings to offset the risks of obesity, diabetes, and other chronic diseases among children.

## Author contributions

Both authors listed have made a substantial, direct, and intellectual contribution to the work and approved it for publication.

## Conflict of interest

The authors declare that the research was conducted in the absence of any commercial or financial relationships that could be construed as a potential conflict of interest.

## Publisher's note

All claims expressed in this article are solely those of the authors and do not necessarily represent those of their affiliated organizations, or those of the publisher, the editors and the reviewers. Any product that may be evaluated in this article, or claim that may be made by its manufacturer, is not guaranteed or endorsed by the publisher.
